# Staying Connected on the Road: A Comparison of Different Types of Smart Phone Use in a Driving Simulator

**DOI:** 10.1371/journal.pone.0148555

**Published:** 2016-02-17

**Authors:** Jaimie McNabb, Rob Gray

**Affiliations:** Human Systems Engineering, Arizona State University, Mesa, Arizona, United States of America; Beihang University, CHINA

## Abstract

Previous research on smart phone use while driving has primarily focused on phone calls and texting. Drivers are now increasingly using their phone for other activities during driving, in particular social media, which have different cognitive demands. The present study compared the effects of four different smart phone tasks on car-following performance in a driving simulator. Phone tasks were chosen that vary across two factors: interaction medium (text vs image) and task pacing (self-paced vs experimenter-paced) and were as follows: Text messaging with the experimenter (text/other-paced), reading Facebook posts (text/self-paced), exchanging photos with the experimenter via Snapchat (image, experimenter -paced), and viewing updates on Instagram (image, experimenter -paced). Drivers also performed a driving only baseline. Brake reaction times (BRTs) were significantly greater in the text-based conditions (Mean = 1.16 s) as compared to both the image-based conditions (Mean = 0.92 s) and the baseline (0.88 s). There was no significant difference between BRTs in the image-based and baseline conditions and there was no significant effect of task-pacing. Similar results were obtained for Time Headway variability. These results are consistent with the picture superiority effect found in memory research and suggest that image-based interfaces could provide safer ways to “stay connected” while driving than text-based interfaces.

## Introduction

Although we still call them “smart phones,” the devices most people carry today serve as a conglomerate of technological devices including a phone, camera, video camera, GPS, computer and entertainment system with over 1 million applications (app’s) available for download. Having such power in the palm of our hand creates a potentially major source of distraction when we are commuting and indeed it is well documented that the use of smart phones can be a cause of accidents for pedestrians (e.g., [1]), cyclists (e.g., [2]), and, the interest of the present study, drivers (reviewed in [3–4]).

To date, the vast majority of studies on the effects of smart phone use in driving have focused on two device functions: talking on the phone and texting/instant messaging. Research on cell phone use has consistently demonstrated that conversations while driving significantly increase a driver’s reaction time (RT) to events and stimuli—a meta-analysis of 33 studies conducted by Caird and colleagues found a mean increase in RT of .25 s [3]. As the authors acknowledge, this is likely an underestimate of the true effect because in most experiments the attentional demands are unlike those in real driving e.g., drivers are abnormally vigilant to the driving task because they are being observed [5]. A key finding of these studies is that the increase in RT is similar for handheld and hands-free phones. This indicates that the detrimental effects of talking on a cell phone while driving are due to a reduction in the attentional and/or working memory resources devoted to driving rather than the commonly held belief that the impairments are solely due to the physical demands of holding the phone and taking one’s eyes of the road to dial/receive a call. It should be noted, however, recent naturalistic driving studies have provided data inconsistent with these findings i.e., no significant increase in the likelihood of an accident when using a hands-free cell phone while driving (e.g., [6]). Although some studies have reported other (perhaps compensatory) effects talking on a cell phone while driving on driver behavior including increased time headway (TH), decreased speed and poorer lateral control, these effects have not been found consistently [4].

Turning to texting, studies have reported similar (and often greater) detrimental effects on driving performance. In a meta-analysis of 28 studies conducted by Caird and colleagues [4], it was found that texting while driving produces significantly longer brake RTs (BRT), increased collisions, as well as adversely affecting lane position, speed and headway regulation. These effects were found both when the driver was typing and reading text messages. A similar pattern of performance degradation was also found regardless of what phone interface was used to text message e.g., participants using either a touch-screen interface (lacking tactile feedback) or a hard numeric keypad interface [7]. Additionally, although slightly less detrimental to overall driving performance, He et al. recently reported that even text messaging via speech-based interfaces negatively impacted driving performance in simulator [8].

In addition to the texting and talking studies Kujala and colleagues have investigated the effects of using a music player and a navigation device on a smart phone during driving [9–10]. Both of these tasks were found to significantly impair driving performance in a simulator (e.g., increases the number of lane excursions) with the effect magnitude depending on the design of the interface (e.g., kinetic vs. button scrolling, grid vs list menu).

As discussed above, smart devices now have many other uses including the main interest of the present study: social networking (e.g., Facebook, Twitter, etc). To date, we have been only able to find one study that has specifically focused on the effect of social networking on driving other than solely texting or instant messaging. Basacik et al. investigated the effect of Facebook use on driving performance in a simulator [11]. In this study, participants were asked to send and receive messages through Facebook’s instant messaging system and update their status. Relative to an only driving control condition, Facebook use resulted in an significant increase in the RT to target stimuli (by 0.4 s on average), increase in the number of lane departures, increased variability of TH, and increased amount of time with eyes off the road. Similar effects were observed for both instant messaging and status updating.

The limited amount of research on the effects of social networking on driving represents an important gap in the literature for two reasons. First, the frequency of this behavior is continuing to increase particularly in younger drivers. A State Farm survey conducted in 2014 [12] reported that between the years 2009–2014 the number of drivers reading social media networks while driving increased from 21 to 41% for the ages 18 to 29 and increased from 9 to 20% across drivers of all ages. Over the same time period, talking on a hand-held phone while driving has decreased. The second important issue is that modern social networking apps include a variety of different methods of interaction that go beyond reading text on a screen and writing text messages. These include exchanging photos (e.g., Snapchat, Instagram) and videos (e.g., YouTube, Vine) which have been shown to have different memory and attentional demands than text (e.g., [13]). For example, there is a well-documented “picture superiority effect” in that memory for images is better than memory for text [14]. Finally, unlike the cell phone use and texting tasks used in most previous driving studies which are typically initiated by someone else, many social networking apps can be either self or other paced (e.g., Facebook). Previous research has shown that for self-paced secondary tasks drivers may adapt their driving behaviors and engage in secondary tasks when a situation is less demanding such as when they are stopped at a red light or before entering “danger zones” [15, 16], however these findings have not be entirely consistent and more research is needed to determine the differences between self and other-paced secondary tasks in driving. For these reasons the effects of social networking on driving may not be directly predictable from the research on cell phone use and texting described above.

The aim of the present study was to compare the effects of four different smart phone tasks on driving performance. In particular, we were interested in two factors: interaction medium (text vs image) and interaction type (self-paced vs experimenter-paced). To achieve this end, drivers were asked to perform four different smart phone tasks (in separate blocks) while also performing a car-following task in a driving simulator: Text messaging with the experimenter (text/experimenter-paced), reading Facebook posts (text/self-paced), exchanging photos with the experimenter via Snapchat (image, experimenter-paced), and viewing updates on Instagram (image, self-paced). As described below, these tasks were designed so that they required a comparable amount of manual interaction and off-road glances. Drivers also performed a baseline driving condition and their performance on the secondary tasks was assessed via post-driving recognition tests. The experiment was designed to test the following predictions:

1. Driving performance would be impaired (e.g., significantly higher BRTs, larger variance in TH) in all four smart phone conditions in comparison to just driving
2. Impairments in driving performance would be significantly greater for the text-based tasks (texting and Facebook) as compared to the image based tasks (Snapchat and Instagram) due to the higher processing demands required for the former [14]
3. Impairments in driving performance would be significantly less for the self-paced tasks (Facebook and Instagram) as compared to the experimenter-paced tasks (texting, Snapchat) because, as found in previous studies, drivers would adapt their behavior to perform the self-paced tasks in less dangerous intervals

## Method

### Participants

Eighteen undergraduates from Arizona State University participated for partial fulfillment of an introductory psychology research requirement. All were native English speakers with normal or corrected-to-normal vision with a valid driver’s license, were right-handed and were smart phone users. Participants ranged in age from 18–22 years (*M* = 20.4). All participants gave informed written consent and the experiment was given ethics approval by the Arizona State University Institutional Review Board (IRB).

### Apparatus

**Fig 1:**
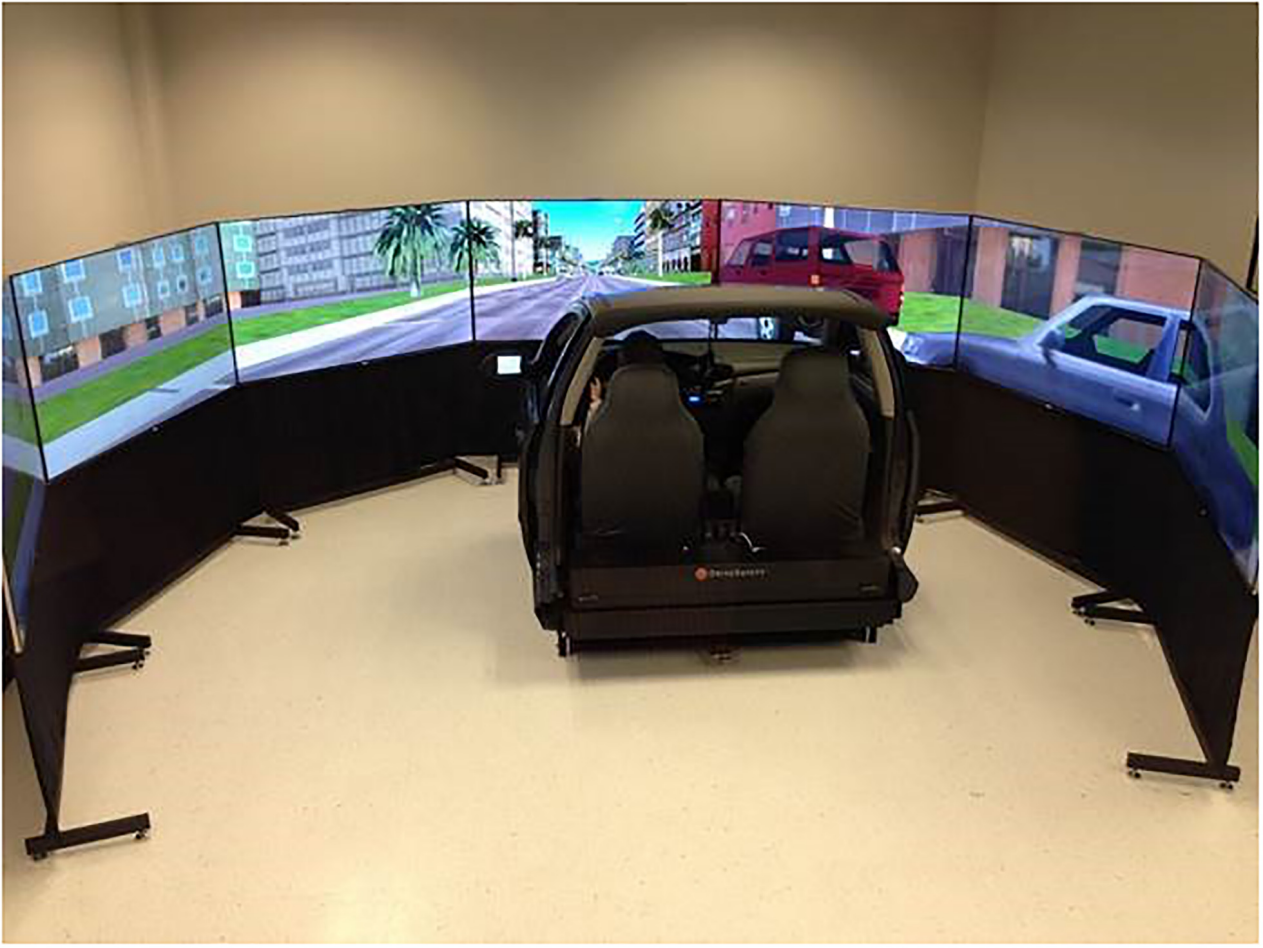
The driving simulator.

The DS-600c Advanced Research Simulator by DriveSafety^™^ was used. As shown in Fig 1, this simulator was comprised of a 300 deg wraparound display, a full-width automobile cab (a Ford Focus) and a motion platform. Tactile and proprioceptive feedback cues were provided via dynamic torque feedback from the steering wheel and vibration transducers mounted under the driver’s seat. The motion platform provided coordinated inertial cues for the onset of longitudinal acceleration and deceleration. The data recording rate was 60 Hz.

During the drive, the participant’s face and right hand (i.e., the one used to hold their phone) were video recorded using a Logitech C920 Webcam.

### Procedure

#### Car following task

The car following task was identical to that used in several previous studies (e.g., [17–19]). Specifically, drivers followed a red lead car on a rural, two-lane road and were instructed to drive in their own lane and not pass the lead car. Drivers were instructed to maintain a 2.0 s time headway (TH) with the lead car. If the drivers followed too far behind the lead car, the words “Speed Up!” would appear in red text on the driver’s display. There was no analogous “Slow Down!” warning so that drivers were free to maintain any TH below 2.0 sec. Drivers were given a 5-min practice drives (with no secondary tasks) to become familiar with the driving simulator and the car following task.

The lead car was programmed to unpredictably (to the driver) change speeds at variable intervals. The lead car traveled between 55 and 65 mph (with an average speed of 60 mph) with its speed determined by a sum of three sinusoids. The lead car was programmed to make 8 unpredictable (to the driver) full stops at a -6 m/s^2^. The behavior of the lead car made it very difficult for the driver to predict when the lead car would speed up, slow down, or stop; creating multiple possible rear-end collision situations. Intermittent opposing roadway traffic was included to more closely simulate real-world rural driving conditions. If the participant contacted the lead vehicle (i.e., crashed) an audio file of a crash sound was presented for a duration of 500 msec and lead vehicle disappeared from the screen.

Each driver completed 5 driving tracks corresponding to the four smart phone tasks plus the baseline driving condition. Each track had 10 unpredictable full stops of the lead car, and required roughly 6–7 minutes to complete. The location of the stops was randomly varied across tracks. Similar to our previous studies (e.g., [17]) drivers always performed the baseline condition first. The order of the 4 smart phone conditions was partially counterbalanced across participants. In particular, we ensured that each of the 4 tasks occurred first and last for an equal number of participants. Participants rested for 5 min between conditions to minimize simulator sickness and fatigue.

#### Smart phone tasks

After the practice and baseline driving condition participants were next given instructions about the four smart phone tasks. For all tasks, participants used their own smart phone and were informed that they would be given a memory test about the content of the task after the driving was complete. The tasks were designed to produce comparable levels of manual interaction with the phone and off-road glances and these variables were analyzed as described below. The four tasks were as follows.

**Texting:** In this condition, participants were told to imagine that they were selling their own car on Craigslist and a potential buyer was contacting them to ask questions. The experimenter then sent several questions (e.g., “what the make, model and year of your vehicle, how long have you owned it?) to which the participant was required to write a text response as they typically would respond. Participants were sent a text once per minute.

**Facebook:** In this condition, participants were required to scroll through and read the updates from a Facebook account made up by experimenters. All of the updates were text only and included no images. Examples were: “A day without sunshine is like, well, night” and “Whatever you are, be a good one.” There were a total of 100 possible updates.

**Instagram:** In this condition, participants were required to scroll through and view the updates on an Instagram account posted by the experimenters. All of the updates were images with no text and there was 120 in total.

**Snapchat:** In this condition, participants were required to view an image sent by the experimenter and then choose an image from the photo album on their phone that matched in some way (e.g., sending a picture of a dog in response to being sent a picture of a dog). Prior to the beginning of the experiment, participants were sent 12 images by the experimenter to save on their phone. Participants were sent an image once every 30 sec.

After each condition, participants completed the NASA-TLX workload questionnaire as well as a recognition test. The recognition tests involved 10 images (for the Snapchat and Instagram conditions) or 10 written statements (for the Facebook and texting conditions) in which only 5 items were actually used in the experiment and 5 were not. After completing the 5 tracks participants filled out a demographics sheet, were debriefed and were given credit for their participation.

### Data Analysis

To assess driving performance two dependent variables that have been shown to be sensitive to distraction in previous studies, BRT and TH variability were used. To compare the visual and manual demands of the smart phone tasks we calculated total on-phone glance time and total time in which the participant’s thumb was in contact with their phone for each condition. These data were analyzed using separate One-Way Repeated Measures ANOVAs with task condition (baseline, texting, Facebook, Instagram and Snapchat) as the factor. Planned comparisons were used to test hypotheses (ii) and (iii) listed above. One-Way Repeated Measures ANOVAs were also performed for the NASA-TLX and the recognition test data.

For all results reported, statistical significance is set at *p<*.*05*. Effect sizes were calculated using partial eta squared (*η*_*p*_^*2*^) for ANOVAs and Cohen's *d* for *t*-tests for all significant findings.

## Results

### Brake Reaction Times (BRT)

**Fig 2:**
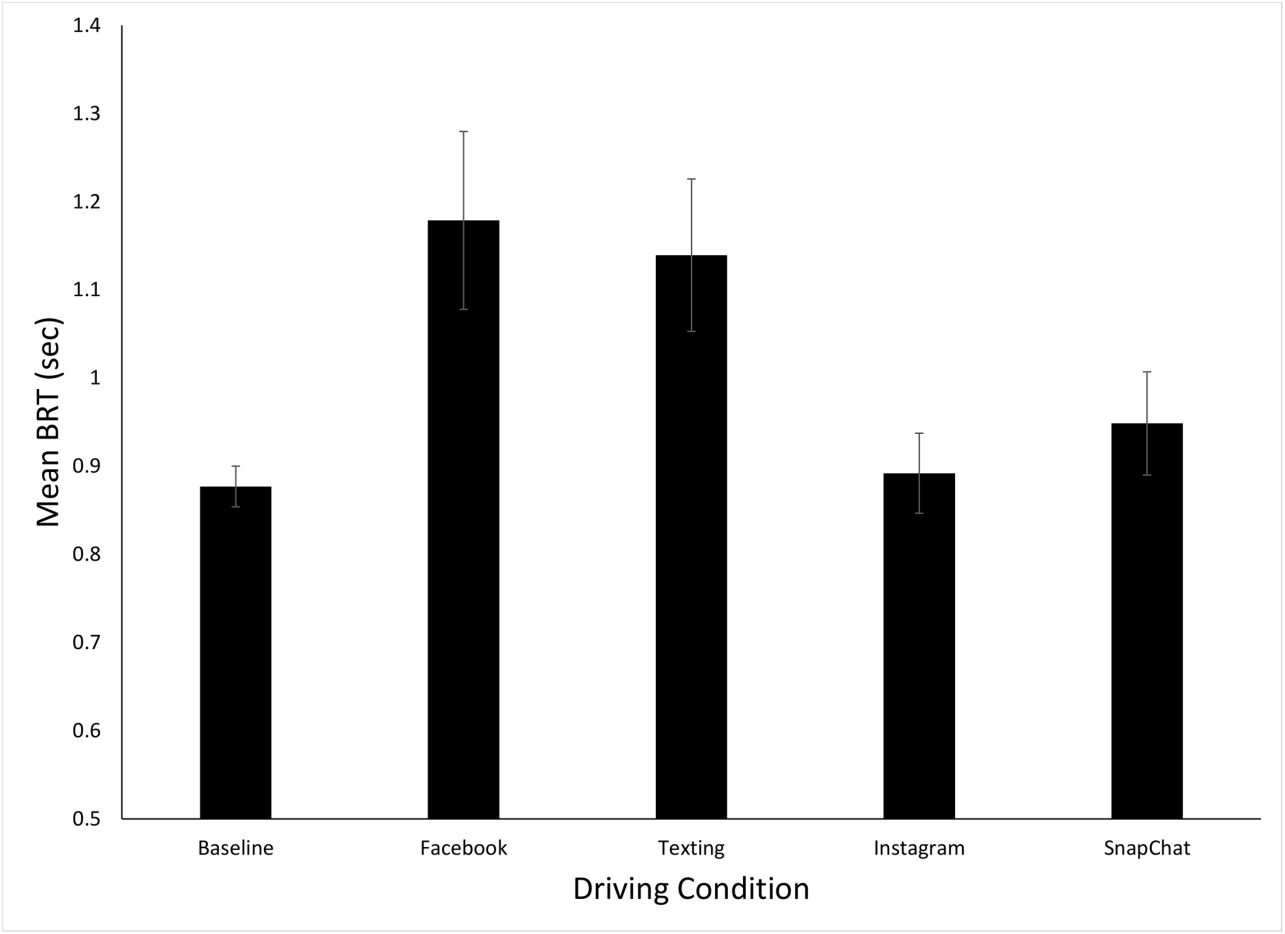
Mean brake reaction times (BRT) for the five different driving conditions. Error bars are standard errors.

Fig 2 shows the mean BRTs for the five different driving conditions (data in S1 Appendix). The one-way ANOVA performance on these data revealed a significant effect of condition, *F*(4, 68) = 5.93, *p<*.*001*, *η*_p_^2^ = .26. The first planned contrast indicated that the combined BRT for the text-based tasks (texting and Facebook, Mean = 1.16 s) was significantly larger than the combined BRT for the image based tasks (Snapchat and Instagram, Mean = .92 s), *t*(17) = 3.47, *p =* .*001*, *d* = 0.75. The second planned contrast revealed that the combined BRT for the self-paced tasks (Facebook and Instagram, Mean = 1.04) was not significantly different that the combined mean for the experimenter-paced tasks (texting, Snapchat, Mean = 1.04), p>.9. Finally, post-hoc t-tests (with Bonferroni correction, p = 0.0125) were used to compare each of the BRT in each of the phones conditions with baseline driving. These tests revealed that, in comparison to the baseline condition (Mean = 0.88 s), the mean BRT was significantly higher in the Facebook (Mean = 1.18 s, t(17) = 3.30, p = 0.004, d = 1.2) and texting (Mean = 1.14 s, t(17) = 3.40, p = 0.003, d = 1.15) conditions. The mean BRTs were not significantly different from the baseline in either the Instagram (Mean = 0.89 s) or Snapchat (Mean = 0.95 s), p’s both >0.25, d’s both <0.25. Finally, there was no significant effect of condition order on BRT, p>0.25.

### Time Headway (TH) variability

**Fig 3:**
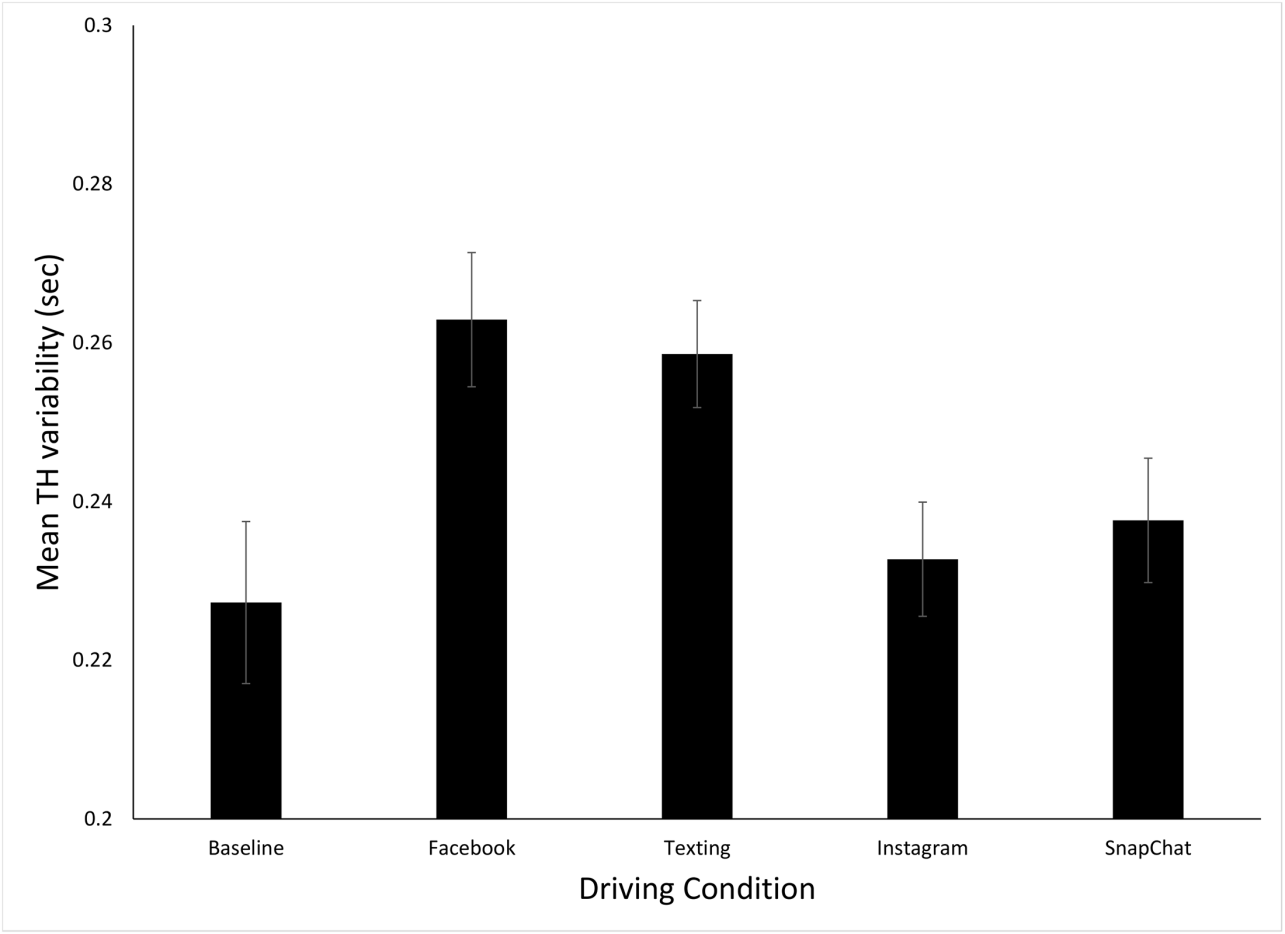
Mean time headway (TH) variability for the five different driving conditions. Error bars are standard errors.

Fig 3 shows the mean TH variability for the five different driving conditions (data in S1 Appendix). The one-way ANOVA performance on these data revealed a significant effect of condition, *F*(4, 68) = 5.02, *p<*.*001*, *η*_p_^2^ = .23. The first planned contrast indicated that the combined TH variability for the text-based tasks (texting and Facebook, Mean = .26) was significantly larger than the combined TH variability for the image based tasks (Snapchat and Instagram, Mean = .24), *t*(85) = 3.2, *p =* .*002*, *d* = 0.8. The second planned contrast revealed that the combined TH variability for the self-paced tasks (Facebook and Instagram, Mean = .24) was not significantly different that the combined mean for the experimenter-paced tasks (texting, Snapchat, Mean = .24), p>.9. The post-hoc tests revealed that, in comparison to the baseline condition (Mean = .23), the TH variability was significantly higher in the Facebook (Mean = .26, t(17) = 2.97, p = 0.008, d = .71) and texting (Mean = .26, t(17) = 3.02, p = 0.007, d = 74) conditions. The mean TH variability was not significantly different from the baseline in either the Instagram (Mean = .23) or Snapchat (Mean = .24), p’s both >0.4, d’s both <0.2. Finally, there was no significant effect of condition order on TH Variability, p>0.4.

### Gaze and Manual Interaction Behavior

The mean total on-phone glance times were 135.9 (SD = 26.0), 130.9 (SD = 30.1), 121.7 (SD = 27.2), and 146.4 (SD = 28.0) sec for the Facebook, texting, Instagram and SnapChat conditions respectively. The one-way ANOVA performance on these data revealed a non-significant effect of condition, *p*>0.1, *η*_p_^2^ = .08. The mean total thumb-phone contact times were 58.9 (SD = 10.3), 62.0 (SD = 16.7), 57.8 (SD = 19.3), and 54.2 (SD = 11.5) sec for the Facebook, texting, Instagram and SnapChat conditions respectively. The one-way ANOVA performance on these data revealed a non-significant effect of condition, *p*>0.25, *η*_p_^2^ = .07

### Questionnaire Data

Table 1 shows the means for mental, physical and temporal demand, as well as task performance, required effort and overall frustration dimensions of the NASA-TLX questionnaire. There was a significant effect of task condition on perceived physical demand [*F*(4, 68) = 5.89, *p = <0*.*001*, *η*_p_^2^ = .26] and perceived mental demand [*F*(4, 68) = 3.06, *p = 0*.*022*, *η*_p_^2^ = .15]. As can be seen in Table 1, the baseline condition was rated as easier than all of the other four conditions. There was no significant effect of task condition for any of the other NASA-TLX dimensions.

**Table 1:** Mean task-load response ratings captured from the NASA-TLX form. All responses are based on a 1–21 increment scale with 1 being very low and 21 being very high.

NASA TLX MEASURES						
Condition	Mental Demand	Physical Demand	Temporal Demand	Performance	Effort	Frustration
Baseline	9.06	4.11	8.83	10.50	10.22	7.61
Facebook	12.72	7.50	10.28	11.28	11.22	9.67
Texting	12.28	8.61	10.28	10.33	11.11	7.78
Instagram	11.33	8.39	10.00	10.50	10.56	8.17
Snapchat	11.24	8.06	9.41	9.00	9.41	8.24

A signal detection analysis was completed for each participant’s four recognition tests and d-prime (d’) values were calculated. As can be seen in Table 2, the means for all participants in each of the four task conditions are similar. A one-way ANOVA performed on the mean d’ values revealed a non-significant effect of condition, p>0.05. This non-significant effect implies that the tradeoff between the driving and phone tasks was similar for all conditions.

**Table 2:** Mean d’ (dprime scores) for each of the 4 recognition task conditions.

Recognition Tests Signal Detection Measures	
Condition	d'
Facebook	3.49
Texting	4.83
Instagram	3.57
Snapchat	3.09

## Discussion

Previous research on smart phone use while driving has primarily focused on phone calls and texting. Drivers are now increasingly using their phone for other activities during driving, in particular social media [12], which have different cognitive demands. The aim of the current experiment was to investigate the effect of four different smart phone tasks on driving performance. In particular, we were interested in two factors: interaction medium (text vs image) and interaction type (self-paced vs other-paced).

Given the consistent negative effects of phone use on driving performance that have been found in previous research (reviewed [3, 4]]) our first hypothesis was that BRTs and TH variability would be significantly elevated in all four smart phone conditions as compared to the baseline driving condition. This was not the case as significant effects on driving performance were only found for the two text-based conditions (Facebook and texting). The significant increase in BRT and TH variability for the text-based conditions are consistent with previous research which has shown both reading and entering text on a phone can impair driving performance [4]. The lack of significant effects for the image-based conditions (and the significant difference between the image-based and text-based conditions found for both BRT and TH variability, which was consistent with our second hypothesis) suggests that interacting with image-based smart phone app’s has different effects on driving performance than text.

Consistent with this idea, previous research has shown that the processing of images has different memory and attentional demands in comparison to text, commonly called the picture superiority effect [14]. This is evidenced by the consistent finding that memory for pictures (e.g., a photo of a car) is better than memory for words (e.g., the word “car”) even when the recall task involves written responses. According to dual coding theory, this effect is due to differences in encoding: pictures are encoded twice (first in a sensory based, visual code then in a symbolic, verbal code) while words are only coded once in a verbal code [14]. These dual codes are thought to be independent and additive, thus, the increasing the likelihood it will be accessed from memory. In the image-based condition in the present study this would have made it easier for participants to remember the images they saw while scrolling in Instagram and the images they have saved in their photo album, thus reducing the demands of the task. istent with this idea.

Contrary to our third hypothesis, there was no significant effect of pacing of the phone task (self vs other) on driving performance. The rationale behind this prediction was previous research which has shown that when drivers engage in self-paced tasks they are more likely to adapt their driving behaviors to engage in the secondary task when conditions were deemed as more “safe,” such as when stopped at a red light or before entering an attention demanding situation [2, 15, 16]. We would argue that the most likely reason for the lack a pacing effect in the present study was the nature of the car-followed task used in our study. The task was a highly dynamic, continuous control task in which there were no clear break periods (e.g., stop lights or stretches of road with no traffic) which the driver could use for the phone tasks. For the entire duration of the trial participants were required to maintain a 2 sec TH with vehicle undergoing regular changes in speed and fairly frequent sudden stops. Therefore, there was very limited opportunity for adaptation of driving behavior. It will be important for future research to investigate whether similar effects occur when self-paced adaption is more feasible.

The present study used the task of car following, a driving behavior that has been modelled in several previous studies, therefore, it is interesting to consider how such models might be used to understand driver distraction in this situation. First, it has been proposed in recent models that car following behavior depends on a driver’s memory for the speed and position at a previous time period (e.g., [20–22]). Since it well know that memory is strongly linked to attention (review in [23]], it is likely that distraction from using a smart phone (which takes a driver’s attention away from the road) will alter this specific parameter of the model. Second, it has been shown that traffic forecasting information providing by intelligent transportation systems can have a direct effect on car following behavior [24], therefore, it would be interesting to investigate whether drivers might use such information to mitigate the effects of distraction (e.g., by deciding when to engage in smart phone tasks). Finally, since most car following models used parameters such as time headway, speed and reaction time (e.g., [25] that were shown to be influenced by distraction in the present study, it will be important for future research to attempt to explain and predict the effects of distraction on car following by directly applying such models.

The findings of the present experiment were limited by certain constraints imposed through the simulation paradigm [5]. First, the drivers in the present experiment were fully expecting the lead car to stop suddenly, so driver responses recorded in this simulation were likely faster than can be expected in a real driving situation. However, it is reasonable to expect that the relative differences in BRT between the different conditions would be the same in real driving. Nonetheless, this needs to be tested empirically. Second, although we attempt to match the smartphone tasks in terms of demands, it is possible that the differences between conditions were not solely due to the differences in interaction medium and pace between conditions. Future research is needed in which warnings occur at a much lower frequency.

On a practical level, the present findings suggest that image-based interfaces may provide a relatively safer ways for people to “stay connected” (e.g., interact with social media, instant message friends, etc.) while driving as compared to text-based interfaces– a function that drivers increasingly want in their vehicle [12]. In the present study, viewing image updates and exchanging images with another person on a smart phone did not have any significant effects on driving performance. Since an auditory analog of the picture superiority effect has also been found (e.g., a ringing sound is remembered better than the word “ringing”, [26], it will be interesting for future research to investigate whether earcons [27] have a similar advantage over talking over a phone in terms of driver distraction.

## Supporting Information

S1 Appendix Driving Performance Data. (DOCX)
