# Supplementary material for: Staying Connected on the Road: A Comparison of Different Types of Smart Phone Use in a Driving Simulator
Source: PLoS One. 2016 Feb 17;11(2):e0148555. doi: 10.1371/journal.pone.0148555 (PMC4757568; doi:10.1371/journal.pone.0148555)
Supplement: S1 Appendix — (DOCX) [file pone.0148555.s001.docx]

**S1 Appendix. Driving Performance Data.**

| BRT |  |  |  |  |  |
| --- | --- | --- | --- | --- | --- |
| subject number | Baseline | Facebook | Texting | Instagram | SnapChat |
| 1 | 0.863358 | 1.25401 | 1.011476 | 0.962927 | 0.865 |
| 2 | 0.846684 | 1.100017 | 0.900027 | 0.600018 | 0.783353 |
| 3 | 0.83612 | 1.020859 | 1.063912 | 0.9466 | 0.808357 |
| 4 | 0.65534 | 0.86421 | 0.973921 | 0.725775 | 1.009547 |
| 5 | 0.900022 | 0.793354 | 0.856002 | 0.816683 | 0.836783 |
| 6 | 0.956534 | 1.252108 | 1.178 | 1.134345 | 1.175025 |
| 7 | 0.779735 | 0.855568 | 1.009549 | 0.861133 | 0.81668 |
| 8 | 0.954185 | 0.814002 | 1.250022 | 1.022243 | 0.8605 |
| 9 | 0.970034 | 1.932247 | 1.24633 | 0.733227 | 0.71668 |
| 10 | 1.1025 | 1.520868 | 1.477813 | 1.26431 | 1.141233 |
| 11 | 0.925017 | 0.956343 | 1.153368 | 0.873783 | 0.583345 |
| 12 | 0.833356 | 1.036147 | 1.081506 | 0.714603 | 0.88335 |
| 13 | 0.833353 | 1.311931 | 0.994543 | 0.890012 | 0.994543 |
| 14 | 0.773346 | 0.881274 | 0.885666 | 0.747237 | 0.837416 |
| 15 | 0.91359 | 1.075025 | 1.062325 | 0.626684 | 1.008355 |
| 16 | 0.85835 | 1.011208 | 1.030746 | 1.183359 | 1.13679 |
| 17 | 0.822227 | 1.066692 | 0.866813 | 0.83335 | 1.733373 |
| 18 | 0.962808 | 2.47226 | 2.46672 | 1.12057 | 0.88336 |
|  |  |  |  |  |  |
|  |  |  |  |  |  |
| TH Var |  |  |  |  |  |
|  | Baseline | Facebook | Texting | Instagram | SnapChat |
| 1 | 0.118 | 0.201908 | 0.212111 | 0.189914 | 0.234847 |
| 2 | 0.256 | 0.2765 | 0.262284 | 0.231196 | 0.26191 |
| 3 | 0.202946 | 0.226437 | 0.241846 | 0.2213 | 0.2711 |
| 4 | 0.235925 | 0.245794 | 0.2422 | 0.198619 | 0.2115 |
| 5 | 0.234 | 0.311752 | 0.272299 | 0.2223 | 0.262836 |
| 6 | 0.204864 | 0.2934 | 0.223751 | 0.225901 | 0.224546 |
| 7 | 0.259934 | 0.285203 | 0.262179 | 0.2124 | 0.271016 |
| 8 | 0.257764 | 0.289803 | 0.275677 | 0.24768 | 0.1956 |
| 9 | 0.2567 | 0.25336 | 0.234996 | 0.272867 | 0.253979 |
| 10 | 0.26209 | 0.198193 | 0.2678 | 0.221936 | 0.2034 |
| 11 | 0.223845 | 0.224149 | 0.273866 | 0.222864 | 0.293188 |
| 12 | 0.296347 | 0.302 | 0.316531 | 0.254 | 0.2657 |
| 13 | 0.19458 | 0.241694 | 0.266484 | 0.196685 | 0.2046 |
| 14 | 0.2567 | 0.238546 | 0.208104 | 0.22209 | 0.167008 |
| 15 | 0.260401 | 0.255613 | 0.242782 | 0.266608 | 0.261135 |
| 16 | 0.156813 | 0.302005 | 0.2665 | 0.2456 | 0.237083 |
| 17 | 0.195857 | 0.2987 | 0.2887 | 0.221958 | 0.24645 |
| 18 | 0.218002 | 0.287393 | 0.296269 | 0.315167 | 0.211288 |
